# Structural Basis for Dual-Inhibition Mechanism of a Non-Classical Kazal-Type Serine Protease Inhibitor from Horseshoe Crab in Complex with Subtilisin

**DOI:** 10.1371/journal.pone.0018838

**Published:** 2011-04-26

**Authors:** Rajesh T. Shenoy, Saravanan Thangamani, Adrian Velazquez-Campoy, Bow Ho, Jeak Ling Ding, J. Sivaraman

**Affiliations:** 1 Department of Biological Sciences, National University of Singapore, Singapore, Singapore; 2 Department of Microbiology, National University of Singapore, Singapore, Singapore; 3 Institute of Biocomputation and Physics of Complex Systems (BIFI), Universidad de Zaragoza, Zaragoza, Spain; 4 Fundacion ARAID, Diputacion General de Aragon, Zaragoza, Spain; 5 Department of Pathology, Center for Biodefense and Emerging Infectious Diseases, University of Texas Medical Branch, Galveston, Texas, United States of America; University of Oulu, Germany

## Abstract

Serine proteases play a crucial role in host-pathogen interactions. In the innate immune system of invertebrates, multi-domain protease inhibitors are important for the regulation of host-pathogen interactions and antimicrobial activities. Serine protease inhibitors, 9.3-kDa CrSPI isoforms 1 and 2, have been identified from the hepatopancreas of the horseshoe crab, *Carcinoscorpius rotundicauda.* The CrSPIs were biochemically active, especially CrSPI-1, which potently inhibited subtilisin (Ki = 1.43 nM). CrSPI has been grouped with the non-classical Kazal-type inhibitors due to its unusual cysteine distribution. Here we report the crystal structure of CrSPI-1 in complex with subtilisin at 2.6 Å resolution and the results of biophysical interaction studies. The CrSPI-1 molecule has two domains arranged in an extended conformation. These two domains act as heads that independently interact with two separate subtilisin molecules, resulting in the inhibition of subtilisin activity at a ratio of 1:2 (inhibitor to protease). Each subtilisin molecule interacts with the reactive site loop from each domain of CrSPI-1 through a standard canonical binding mode and forms a single ternary complex. In addition, we propose the substrate preferences of each domain of CrSPI-1. Domain 2 is specific towards the bacterial protease subtilisin, while domain 1 is likely to interact with the host protease, Furin. Elucidation of the structure of the CrSPI-1: subtilisin (1∶2) ternary complex increases our understanding of host-pathogen interactions in the innate immune system at the molecular level and provides new strategies for immunomodulation.

## Introduction

Serine proteases play an important immunomodulatory role in host-pathogen interactions. Invertebrates lack an adaptive immune system that recognizes and remembers specific pathogens [1]. As an evolutionarily conserved and ancient defense strategy, the innate immune system responds instantaneously to invading pathogens in a non-specific manner. The innate immune system in the horseshoe crab, *Carcinoscorpius rotundicauda*, comprises serine protease cascades that are similar to the blood coagulation, melanization and complement systems [2]. Horseshoe crab hemocytes contain granules filled with several serine protease zymogens. Upon mechanical injury or pathogen invasion, the granules are released into the extracellular milieu by exocytosis. Furthermore, clotting enzymes, in their precursor forms, are activated by a serine protease cascade that is triggered by bacterial endotoxins [3], [4]. Several serine protease zymogens, including proclotting enzymes, Factor B and Factor C, are associated with the hemolymph coagulation system. The subsequent formation of the coagulation plug prevents further entry of pathogens [5], [6].

In parallel, a large number of protease inhibitors are also expressed in hemocytes with varying specificities, which target the proteases of different microorganisms [7], including the serine proteases like elastase, subtilisin and protease K. These inhibitors belong mainly to the Kazal, Kunitz and α2-macroglobulin families and they use a lock-and-key mechanism with their reactive site loops to mechanistically bind the active sites of the target proteases [8]. It has been speculated that these inhibitors might participate in the regulation of the hemolymph coagulation cascade [9], [10]. During our recent work on subtractive ESTs (expressed sequence tags) from the *Carcinoscorpius rotundicauda* that was infected with *Pseudomonas aeruginosa*, two isoforms of the non-classical Kazal-type inhibitor, CrSPI, each of 9.3 kDa, were discovered [9]. The CrSPI-1 and CrSPI-2 (GenBank Accession numbers DQ090491 and DQ090492, respectively) isoforms share 97% sequence identity. Both isoforms are biochemically active. In our earlier study [9], we have tested representative microbial serine proteases such as proteinase K and subtilisin, in comparison with eukaryotic serine protease, trypsin. While trypsin was inhibited only by CrSPI-2, subtilisin was found to be most susceptible for inhibition by CrSPI-1, with Ki of 1.4×10^−9^ M. Hence subtilisin was used to maximize the exploration of the activity of CrSPI-1. Moreover subtilisin is secreted in large amounts by many bacillus species like *Bacillus subtilis*. *B. subtilis* thrives in the natural habitat (estuarine muddy swamp) of the horseshoe crab (*C. rotundicauda*), although there is no report that *B. subtilis* is a pathogen of *C. rotundicauda*.

Serine protease inhibitors in the plasma have been known to perform dual functions: (i) regulation of the activity of endogenous serine proteases and (ii) inhibition of microbial serine proteases as a host immune defense against invading pathogens [11]. For instance, the human lympho-epithelial Kazal-type inhibitor (LEKTI) plays an important role in epithelial tissue homeostasis through the regulation of trypsin, and dysfunction of LEKTI has been implicated in Netherton syndrome [12]. Furthermore, the Limulus serpins, LICI-1, LICI-2 and LICI-3, from the horseshoe crab are known to regulate serine proteases in the pathogen-induced coagulation cascade [13]. CrSPI may possibly downregulate the blood coagulation serine enzymes, especially in view of helping the host to avoid random activation of coagulation under normal conditions. However, this remains a speculation until future tests are carried out.

The Kazal family is 1 of 18 families of serine protease inhibitors. Since the 1980′s, the structures of several members of this family have been reported [14]. The Kazal family is mainly divided into two groups: the classical and the non-classical inhibitors [15]. The positions of the cysteine residues that form disulfide bonds differ among the classical and non-classical Kazal-type inhibitors [16]. The classical group is best represented by the pancreatic secretory trypsin inhibitor and the ovomucoids. Furthermore, the non-classical Kazal inhibitors are divided into group I and group II. The characteristic feature of group I inhibitors is that the disulfide bond between the first and fifth cysteine residues is shifted towards the C-terminus compared to the respective residues of a classical Kazal inhibitor, like OMTKY3.

Both domains 1 and 2 of CrSPI-1 share sequence homology with the non-classical group I Kazal-type inhibitors. Other representatives of the non-classical group I Kazal-type inhibitors are the *Anemonia* elastase inhibitor, Crayfish inhibitor [17], *Ciona* trypsin inhibitor [18] and *Galleria* trypsin inhibitor [19]. The non-classical group II inhibitors have a cystine-stabilized α-helical motif (CSH motif) composed of an α-helix that spans the Cys-X_1_-X_2_-X_3_-Cys portion and is cross-linked by two disulfide bridges. Representatives of this group are the Leech Derived Trypsin inhibitor (LDTI-C) [20], Bdellin B-3 from the leech *Hirudo nipponia* [21], Rhodniin from the blood-sucking insect *Rhodnius prolixus* [22], the thrombin inhibitor Dipetalogastin from the bloodsucking insect *Dipetalogaster maximus* and Ascidian Trypsin Inhibitor [23] from the sea squirt.

Although several structures have been reported for Kazal-type inhibitors, a structure has not been reported for a protease inhibitor from the hemolymph of an ancient arthropod. The horseshoe crab has been dubbed a “living fossil” with several hundred million years of evolutionary success, indicating that it harbors a formidable antimicrobial system. Therefore, it is conceivable that the CrSPI from the *Carcinoscorpius rotundicauda*, which thrives in microbiologically-challenging habitat, would exhibit unique antimicrobial properties. Moreover, there are no crystal structures available for a non-classical group I Kazal-type inhibitor, except for the solution structure of the *Anemonia* elastase inhibitor from the sea anemone [15]. Here, we report the crystal structure of the two-headed non-classical Kazal-type group I inhibitor CrSPI-1 in complex with its cognate protease, subtilisin, at a stoichiometric ratio of 1∶2 (CrSPI-1 to subtilisin) and refined up to 2.6 Å resolution. The reactive site loops of both domains of CrSPI-1 occupy the substrate binding pockets of subtilisin. Furthermore, based on our structural and biophysical interaction studies, we propose that domain 2 of CrSPI-1 is a more specific and potent inhibitor of subtilisin, whereas domain 1 likely interacts with CrFurin (*Carcinoscorpius rotundicauda* Furin), a subtilisin homolog of the host that belongs to the family of Furins or kexins, which are known to be involved in the processing of protein precursors, including many immune proproteins [24]. Earlier, we have proposed that CrSPI-1 may act as an “on-off” regulatory switch in the modulation of antimicrobial activities while maintaining homeostasis of host proteases [9]. Our present findings provide a new structural insight into host-pathogen interaction. Our delineation of the bioactive sites of the two domains of CrSPI-1, which could differentiate between the bacterial and host proteases, might provide an impetus for the development of new strategies for novel antimicrobial drugs and immunomodulators.

## Methods

### Expression, purification, crystallization and data collection

The CrSPI-1 gene was cloned and expressed in *E. coli* with the pET32-EkLIC system (Novagen) using the following primers: Forward 5′ ACG GAC GAC GAC AAG ATG TGT CCT CAT ACT TAC AAA 3′ and Reverse 5′ ACG GAG GAG AAG CCC GGT TTA CAA GCA AGC TTC TAG TGG 3′ [25]. The expressed protein contained a thioredoxin tag, a His-tag and an enterokinase cleavage site. The recombinant CrSPI-1 was overexpressed at 37°C from a single colony picked from an agar plate. The culture was induced with 300 mM isopropyl 1-thio-D-galactopyranoside for 4 h to an OD_600nm_ of 0.6. Cells were then harvested by centrifugation (9000 g, 20 min, 4°C) and sonicated. The protein was purified using Nickel-NTA affinity beads (Qiagen) with Phosphate Buffered Saline (PBS) at pH 7.4 and 10 mM β-mercaptoethanol (2BME). The protein was eluted in 300 mM imidazole. The thioredoxin tag was cleaved through a 2-h incubation with enterokinase (Sigma). The complex was prepared by mixing CrSPI-1 with subtilisin Carlsberg obtained from *Bacillus licheniformis*, (Sigma) in an approximate molar ratio of 1∶2 (inhibitor to enzyme) and incubated for 1 h at 37°C. The complex was purified using a Superdex-75 gel filtration column, and fractions were pooled and concentrated up to 20 mg/ml. Crystallization screens were performed using the hanging drop vapor diffusion method with Hampton Research Crystal Screen kits I and II and JB Screens (Jena BioScience, Germany) at room temperature. The initial crystallization conditions were further optimized using a grid screen by varying the concentration of the precipitant. Plate-like diffraction quality crystals were obtained from 11% (w/v) Polyethylene Glycol 20000 in 0.1 M 2-(N-morpholino) ethanesulfonic acid (MES) at pH 6.5. The crystallization solution was supplemented with 25% glycerol, which acts as a cryo-protectant. A complete dataset was collected in the X29-A synchrotron beamline at Brookhaven National Laboratories, USA. The data collection and refinement statistics are provided in Table 1.

**Table 1:** Crystallographic data and refinement statistics.

**Data collection** #	
Unit cell parameters(Å, °)	a = 73.8, b = 65.1, c = 111.9α = 90, β = 95.44, γ = 90
Space group	P2_1_
Resolution range (Å)	50-2.6 (2.64-2.60)
Wavelength (Å)	0.9600
Observed *hkl*	205529
Unique *hkl*	32884
Completeness (%)	99.8 (99.3)
Redundancy	6.3 (5.3)
Overall *I/σI*	7.2 (3.3)
aR_sym_ (%)¶	10 (34)
**Refinement and quality of model**	
*Resolution range (Å)	15-2.6
bR_work_ (no. reflections)	0.21 (24818)
cR_free_ (no. reflections)	0.24 (1533)
Root mean square deviation	
Bond length (Å)	0.009
Bond angle (°)	1.52
Ramachandran plot	
Favored region (%)	93.9
Allowed regions (%)	5.0
Generously allowed region (%)	1.1
Disallowed regions (%)	0.0
dAverage B-factors (Å^2^)	
CrSPI-1 (no. of atoms)	57.2(514)
Subtilisin (no. of atoms)	32.4(5760)
Overall protein atoms (no. of atoms)	37.4 (6274)
Waters (no. of atoms)	35.5 (168)

a R_sym_ =  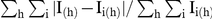 where I_i(h)_ and I_(h)_ are the i^th^ and mean measurement of reflection h.

b R_work_  =  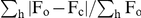, where F_o_ and F_c_ are the observed and calculated structure factor amplitudes of reflection h.

c R_free_  =  as for R_work_, but approximately 8% of the total reflections chosen at random and omitted from refinement.

d Individual B-factor refinement were carried out.

*Reflections greater than I>σI where used in the refinement.

# Values in the parentheses are corresponding to the highest resolution shell.

¶ Indicates the moderate quality of data.

### Structure solution and refinement

The structure of the recombinant CrSPI-1: subtilisin complex was solved by the molecular replacement method with Molrep [26] using the subtilisin Carlsberg coordinates (PDB code 1SCA). The initial R-factor was 47% and subsequent refinement was performed with Refmac [27] and CNS [28]. Non-crystallographic symmetry (NCS) restraints were used for subtilisin molecules during the refinement in CNS. When the R-factors were close to 40, the calculated electron density map allowed us to build the CrSPI-1 molecule. Several cycles of refinement in CNS with alternating model building for the inhibitor complex was carried out using the programs O [29] and Coot [30]. It was noticed that the B-factors of CrSPI-1 molecule is higher, might indicate its reduced occupancy; however, it is well defined in the electron density map (Figure 4C). The overall geometry of the final model was analyzed by MolProbity [31] which showed 93.9% of the residues in the favored region and no residue in the disallowed region (Table 1).

### Isothermal Titration Calorimetry (ITC)

ITC studies were performed using the MicroCal VP-ITC and MicroCal ITC-200 instruments. CrSPI-1 at a concentration of 0.2 mM in PBS at pH 7.4 and 10 mM BME, was titrated into a solution of 0.012 mM subtilisin in PBS at pH 7.4 and 10 mM BME in the sample cell with injections of 2 µL (ITC-200) or 10 µL (VP-ITC). Additionally, the VCTEEY peptide, at a concentration of 11 mM in PBS at pH 7.4 and 10 mM BME, was titrated into a 0.063 mM subtilisin solution in PBS at pH 7.4 and 10 mM BME in the sample cell with injections of 1 µL. This peptide was designed based on the reactive site loop sequence of CrSPI-1 that is in the proximity of the active site of subtilisin in the CrSPI-1: subtilisin complex in the crystal structure. The VCTEEY peptide, which contains the RSL residues from P4 to P2′ of CrSPI-1 domain 2, was purchased from Sigma-Aldrich. All experiments were performed at 37°C. Samples were degassed prior to use. Due to dilution across the needle during equilibration, the initial peak was discarded. Data analysis was performed using the MicroCal Origin software. For the rCrSPI-1 experiments, a model considering an interaction with a binding stoichiometric ratio of 1∶2 CrSPI-1: subtilisin (CrSPI-1, the macromolecule with two binding sites for subtilisin is located in the syringe) in terms of the overall association constants or the site-specific intrinsic microscopic association constants was employed [32], [33], whereas for the VCTEEY peptide binding experiments, a model considering a stoichiometric ratio of 1∶1 VCTEEY peptide to subtilisin was employed. Experiments were performed in duplicate. Typical errors were 15–20% for the association constant and 5–10% for the binding enthalpy.

## Results

**Figure 1:**
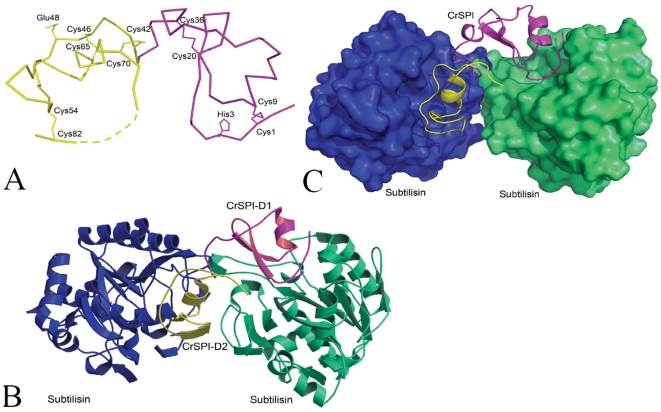
Structure of the CrSPI-(subtilisin)_2_ complex. (A) Shows the Cα trace of CrSPI-1. The disulphide bonds are shown in thick lines along with the residue numbers. Domain-1 is in magenta and domain-2 is in yellow color. The approximate location of the missing residues (residues 74 to 81) is shown as dotted line. The P1 residues His3 and Glu48 of both domains are shown. (B) Ribbon representation of subtilisin molecules are drawn in blue and green color and two domains of rCrSPI are in magenta and yellow color. (C) Shows the CrSPI-1: subtilisin complex. CrSPI-1 is in ribbon representation and subtilisin is in surface representation. These figures were prepared by using PyMol [54] and Molscript [55].

The structure of recombinant CrSPI-1 in complex with subtilisin was solved by the molecular replacement method from a synchrotron dataset. The model was refined to a final R-factor of 0.21 (R_free_ = 0.24) at 2.6 Å resolution with good stereochemical parameters (Table 1). The final refined model consisted of residues from Cys1 to Val73 of CrSPI-1 and Ala1 to Gln274 of subtilisin. Eight residues (Val74-Glu81) at the C-terminus of CrSPI-1 lacked interpretable electron density and were not modeled. There were three subtilisin molecules and one CrSPI-1 molecule in the asymmetric unit. Each CrSPI-1 molecule interacted with two subtilisin molecules, i.e., domain 1 and domain 2 of CrSPI-1 interacted with two independent subtilisin molecules (Fig. 1). The third subtilisin molecule of the asymmetric unit was not in a complex with CrSPI-1. This indicates that free subtilisin molecules were present in the crystallization drop due to the lack of adequate inhibitor molecules.

The conformation of the subtilisin molecules in the complex was similar to that seen in the subtilisin Carlsberg (EC 3.4.21.62) structure and structures of subtilisin complexed with small ligands [34]–[36]. The structure consists of a central seven-stranded parallel β-sheet with two α-helices on one side and a group of four α-helices on the other side of the central β-sheet. The catalytic triad, which consists of Ser220, His64 and Asp32, was located in the substrate-binding cleft. The three subtilisin molecules of the asymmetric unit were identical with an rmsd of 0.1 Å in a pairwise superimposition of 274 Cα atoms. This indicates that there was no significant conformational change of subtilisin molecules upon complex formation with the CrSPI-1 molecule.

### Structure of CrSPI-1

**Figure 2:**
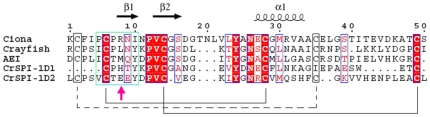
Multiple sequence alignment for the representative members of Kazal-type Non classical group I proteinase inhibitors- Ciona trypsin inhibitor, crayfish inhibitor, Anemonia elastase inhibitor, CrSPI-1 domain 1 and CrSPI-1 domain 2. Secondary structural elements are shown above the sequences. Conserved residues are shaded in red and yellow boxes. The reactive site loop residues of P4-P2′ are represented in a green box. The reactive site residue P1 is shown using an upward pointing magenta arrow. This figure was prepared by the program Espript [56].

The CrSPI-1 molecule comprises two domains: domain 1 from Cys1 to Glu40 and domain 2 from Leu41 to Leu83. Both domains adopted similar secondary structures (domain 1 β1↑β2↓α1β3↑ and domain 2 β4↑β5↓α2). The presence of a central α-helix, α1 (Glu18–Ala24) in domain 1 and α2 (Arg63-Ser68) in domain 2, and an antiparallel β-sheet in each domain are characteristic of the classical Kazal-type inhibitors. However, CrSPI-1 also showed features of a non-classical Kazal-type serine protease inhibitor in that it harbored an unusual pattern of conserved cysteines. There were two intra-domain disulfide bridges in domain 1 (Cys1-Cys19 and Cys8-Cys35) and three in domain 2 (Cys41-Cys70, Cys45-Cys64 and Cys53-Cys82) (Fig. 1A). Using a BLAST search, the sequence identity between CrSPI-1 and the most homologous member of the Kazal family of inhibitors was analyzed. The observed pattern of S-S bridges in CrSPI-1 was more similar to the non-classical Kazal-type group 1 inhibitors from sea anemone and crayfish than to the mammalian and avian inhibitors (Figs. 2 and S2).

A search for topologically similar proteins within the PDB database was performed with the DALI program [37]. There were no structural homologs of full-length CrSPI-1; however, the DALI search with individual domains showed similarity with several Kazal-type inhibitors. The DALI search with domain 1 (residues Cys1-Glu40) against the pdb database showed that it was structurally similar to leech-derived tryptase inhibitor (pdb code 1ldt) with an rmsd of 2.0 Å for 38 Cα atoms (30% sequence identity). This was followed by dipetalin, a Kazal-type thrombin inhibitor (rmsd = 2.0 Å for 37 Cα atoms; 27.5% identity; pdb code 1kma) and an insect-derived double domain Kazal inhibitor, rhodniin, which is a highly specific thrombin inhibitor (rmsd = 1.5 Å for 36 Cα atoms; 35% identity; pdb code 1tbq). Similarly, the structural homology search with domain 2 (Leu41-Leu83) showed several matches with Kazal-type inhibitors. Domain 2 is structurally homologous to turkey ovomucoid third domain (OMTKY3), a subtilisin inhibitor (pdb code 1yu6), with an rmsd of 2.3 Å for 32 Cα atoms with 25% sequence identity. This was followed by the thrombin inhibitor rhodniin (rmsd = 2.3 Å for 31 Cα atoms; 7.5% identity; pdb code 1tbq) and leech-derived tryptase inhibitor (pdb code 1an1), which has an rmsd of 2.4 Å for 29 Cα atoms with 20% sequence identity. Most of the structurally characterized Kazal-type inhibitors contain a single domain, except for rhodniin. Rhodniin is the only structurally characterized Kazal-type inhibitor with two domains in complex with thrombin (1∶1 ratio). Both CrSPI-1 domains show significant structural similarity to the respective domains of rhodniin. However, full-length CrSPI-1 could not be superimposed with full-length rhodniin. This indicates that the relative orientations of the two domains are different. Notably, Rhodniin binds with only a single serine protease (thrombin) whereas CrSPI-1 binds with two serine protease molecules. This might be due to the variations of the reactive site loop residues at the prime side of the substrate binding site, which might dictate the specificity of these inhibitors towards a particular serine protease.

**Figure 3:**
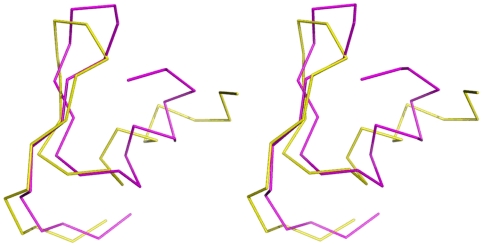
Stereo view of the Cα superimposition of domain-1 (magenta) and domain-2 (yellow) of CrSPI-1. These two domains superimpose with an rmsd of 3.9 Å for 24 Cα atoms. The superimposition was carried out using DALI [37]. This figure was prepared by using PyMol [54].

The comparison of CrSPI-1 domains 1 and 2 revealed that the core regions superimposed with an rmsd of 3.9 Å for 24 Cα atoms (Fig. 3). The sequence alignment showed 27% identical (42% similar) residues between the two domains, including the highly conserved cysteines (Fig. 2). The highly conserved S-S bridges probably maintain the architecture of these domains. The overall fold similarity between these two domains, together with the structural homology with other Kazal-type inhibitors, suggests that all Kazal-type inhibitors evolved from a common ancestral gene via duplication while maintaining divergent amino acid sequences. A study by Merckel *et al.* [38] showed that the similarities between the tertiary structures are indicators of gene duplication.

### CrSPI-1: subtilisin complex

The CrSPI-1: subtilisin complex is a heterotrimer in solution, which was confirmed by gel filtration, non-reducing SDS-PAGE [25] and Isothermal Titration Calorimetry (ITC) experiments (Fig. S3). This is consistent with the observation of a heterotrimer of a 1∶2 ratio of CrSPI-1 to subtilisin in the asymmetric unit of the crystal (Fig. 1). Both domains of CrSPI-1 act as heads that bind with two individual subtilisin molecules. Our earlier work showed that domain 1 alone did not react with subtilisin, whereas domain 2 showed high affinity for subtilisin with a Ki of 2.6 nM [9]. However, the structural studies revealed that although both domains indeed bind to subtilisin, only domain 2 showed tight interactions with subtilisin. We hypothesize some possible reasons for the tighter interaction of domain 2 in the section “Rigidity of the RSL.” The two subtilisin molecules were bound at the opposite ends of the elongated inhibitor molecule and formed a ternary complex. Subtilisin is a monomer in solution; upon formation of a complex with rCrSPI, it becomes a heterotrimer. Rhodniin, a two domain non-classical group II Kazal-type inhibitor from the blood sucking insect *Rhodnius prolixus,* was shown to inhibit thrombin at a 1∶1 ratio by binding of one domain at the active site and the other domain at the exosite [39]. Similarly, the crystal structure of an inhibitor belonging to the potato II inhibitor family, a completely different family of serine protease inhibitors, showed that the two-headed Tomato Inhibitor-II inhibits two subtilisin molecules with a Ki of 9 nM [40]. More recently, a two-headed arrowhead protease inhibitor, which belongs to the Kunitz family of protease inhibitors in a complex with two trypsin molecules, was reported [41]. The present study on the CrSPI-1: subtilisin heterotrimer complex is the first of the non-classical Kazal-type inhibitors that employs a dual-inhibition mechanism to engage two protease molecules with different specificities.

### CrSPI-1 reactive site loop interactions with subtilisin

**Figure 4:**
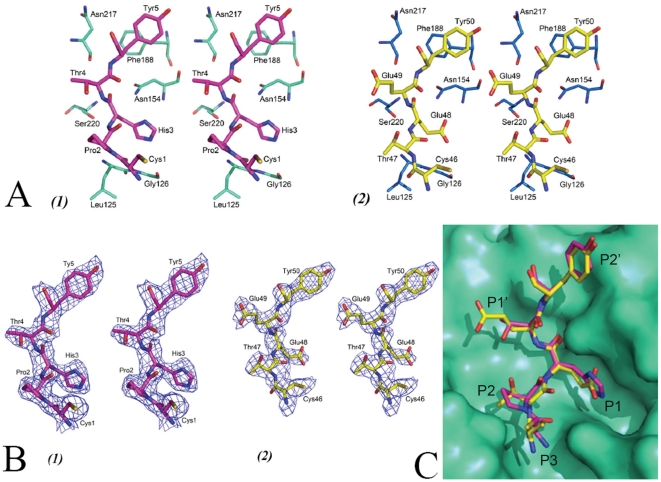
Interactions between CrSPI-1 domains and subtilisin. Stereo view of the interactions between subtilisin and the reactive site loops (RSLs) of (A) (1)domain-1 (magenta) and (2) domain-2 (yellow). For subtilisin and for the CrSPI-1 domains same colors were used as in Fig. 1. (B) Superimposition of the reactive site loops of both domains. (C) Electron density maps. (1) Stereo view of the final *2Fo-Fc* map for the reactive site loop (RSL) region of domain-1 and (2) domain-2 of CrSPI-1 bound to subtilisin. These maps are contoured at a level of 1σ. These figures were prepared by using PyMol [54].

The reactive site loop (RSL) of domain 1 of CrSPI-1 (Cys1 to Lys6) binds at the active site region of subtilisin from P3 (Cys1) to P3′ (Lys6) in a substrate-like manner (Fig. 4A, panel 1). There were 11 hydrogen bonding contacts between the domain 1 RSL and subtilisin. Of these, five hydrogen bonding contacts were mediated by side chains. The main chain amide group of the P1 residue His3 was involved in a hydrogen bonding contact with O^γ^ of the catalytic Ser220 (2.63 Å). The carbonyl O atom of the P1 His3 interacted with the backbone NH group of the active site Ser220 and N^γ2^ of Asn154 of subtilisin (Table S1). In Kazal-type inhibitor complexes, the P1 residue alone makes approximately 50% of the hydrogen bonding interactions with the active site residues [42], [43]. In the CrSPI-1: subtilisin complex, the P1 residue His3 of the CrSPI-domain 1 contributes 5 hydrogen bonding contacts (or 45% of the total hydrogen bonding interactions) with subtilisin. Pro2 occupies the P2 position and makes hydrophobic interactions with subtilisin. In most of the canonical serine proteinase inhibitors, the P3 residue is engaged in a disulfide bond. In CrSPI-1, Cys1 is in the P3 position and engaged in a disulfide bond with Cys19, which is part of the hydrophobic core that consists of Leu125 (subtilisin) and Pro2 (CrSPI). Thr4 is in the P1′ position, and Tyr5 and Lys6 are in P2′ and P3′ positions, respectively. The P2′ residue Tyr5 maintains stacking interactions with Phe188 of subtilisin (Fig. 4A, panel 1).

In domain 2 of CrSPI-1, Glu49 takes the position of His3 in the P1 pocket of domain 1 and makes 8 (73%) of the 11 total hydrogen bonding contacts with subtilisin. The remaining hydrogen bonding contacts with subtilisin are from P1′ Glu49, P2′ Tyr50 and P4 Val45 of domain 2 (Table S2). Similar to the P2′ residue Tyr5 of domain 1, Tyr50 takes the P2′ position in domain 2 and maintains stacking interactions with Phe188 of subtilisin (Fig. 4A, panel 2). The reactive site residues are well defined in the electron density map (Fig. 4C). In addition, the P3 residue Cys46 mediates hydrophobic interactions with the side chain of Leu125 of subtilisin. Overall, the RSL interactions in domain 2 clearly indicate that the P1 residue Glu48 is the primary mediator of the interaction with subtilisin. These observations show that domain 1 may not be a strong inhibitor of subtilisin compared to domain 2. Notably, domain 2 inhibits subtilisin with a Ki of 2.5 nM, whereas CrSPI-1 domain 1 did not inhibit subtilisin [9]. However, the Ki for full-length CrSPI-1 was 1.43 nM.

### Rigidity of the RSL

The conformations adopted by the reactive site loops (RSLs) of both domains of rCrSPI were similar and superimposable with an rmsd of 0.80 Å for the residues from positions P3 to P3′ (Fig. 4B). The RSLs in several families of serine protease inhibitors in many complexes and in different crystal environments adopt similar conformations (Fig. S2). Similar to other members of the Kazal family of inhibitors, the disulfide bonds formed by cysteine residues at the P3 and P5′ positions (Cys1 and Cys8 in domain 1 and Cys46 and Cys54 in domain 2) of CrSPI-1 may hold the RSL in a relatively rigid conformation.

The backbone torsion angles (ψ/ϕ angles) of the RSLs of CrSPI-1 were similar to each other and to other protease inhibitors, such as OMTKY3 and Eglin-C complexed with subtilisin (Table S3). In addition, the torsion angles of the RSL backbones of CrSPI-1 were similar to several structurally unrelated serine protease inhibitor complexes and confirm the canonical binding mode for CrSPI-1 (Table S3).

Superimposition of the active site region of the subtilisin complexes of OMTKY3 and Eglin C with the CrSPI-1: subtilisin complex revealed that different inhibitor loop sequences can be accommodated within the substrate binding site of subtilisin with minimal side chain rearrangement (Fig. S2). Furthermore, it indicates that the RSLs of these inhibitors have a rigid conformation, which is supported by the fact that both main chain and side chain groups of subtilisin remain in the same relative conformation in several complexes and in the uncomplexed form. In domain 2, two out of three S-S bridges (Cys41-Cys70 and Cys45-Cys64) are connected to the central α-helix region and maintain a rigid conformation for the RSL of this domain, unlike the RSL in domain 1. Notably, the presence of three disulfide bonds in domain 2 explains the rigidity, specificity and potency of this domain towards its particular cognate protease subtilisin. It is worth mentioning here that in the case of OMSVP3, engineering an additional disulfide bridge increased the specificity for only one protease, α-chymotrypsin, and decreased the specificity for other proteases [23].

In addition to the S-S bridges, there are three important internal hydrogen bonding contacts that maintain the rigidity of the RSLs in CrSPI-1. These contacts in domain 1 are (1) the P2-P1′ hydrogen bond between the carbonyl oxygen of Pro2 and amide nitrogen of Thr4 of the reactive site loop, (2) the hydrogen bond between Asn18 and Phe21, and (3) the interaction of N^δ2^ of Asn18 with the main chain carbonyl atoms of Pro2 and Thr4 at the P2 and P1′ positions of the RSL. Similarly, in domain 2, the interactions through the side chains of Asn62, Thr47 and Glu49 maintain the rigidity of the RSL. The interactions between Thr47 and Glu49 are similar to the interactions of P2-P1′ of domain 1. In order to reduce the entropic cost of binding, the RSLs of Kazal-type inhibitors and of CrSPI-1 in particular are firmly held in a preferred conformation that is complementary to the substrate-binding site of specific proteases. A rigid conformation of the RSL is thought to prevent proteolytic cleavage of the inhibitor upon interaction with proteases [43].

**Figure 5:**
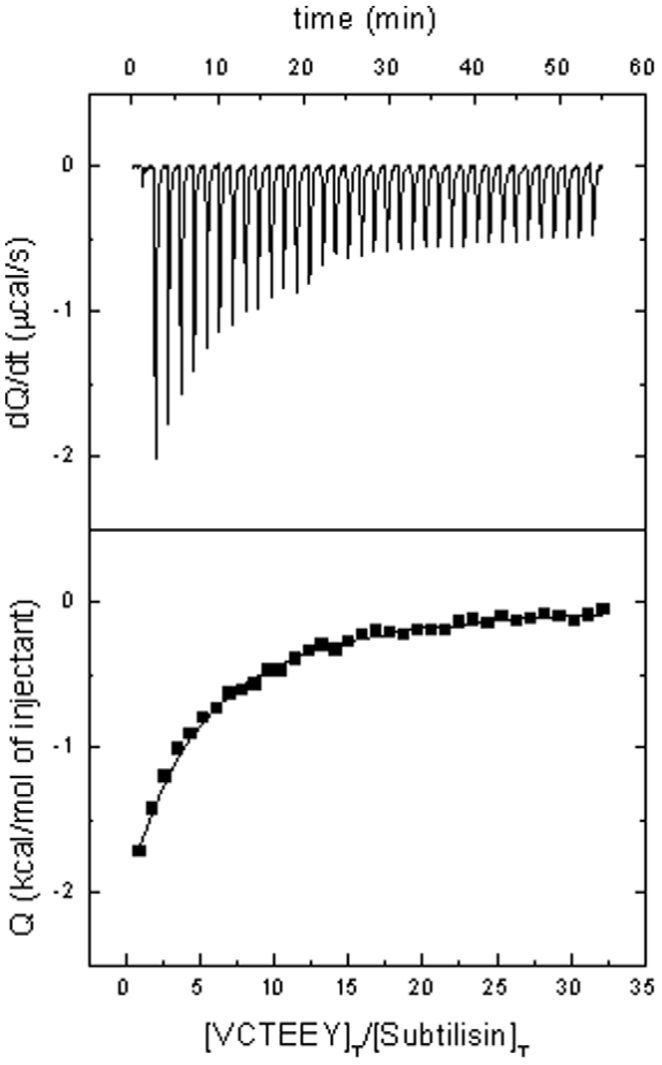
Calorimetric titration for peptide VCTEEY binding to Subtilisin at 37°C. Each peak represents the injection of VCTEEY 11 mM into the calorimetric cell containing 0.063 mM Subtilisin in buffer PBS pH 7.4, 10 mM BME. Injections of 2 µL of ligand solution were performed. The experimental data were fitted considering a model in which Subtilisin binds a single peptide molecule. Non-linear regression analysis provided an association constant of 1.9×10^3^ M^−1^ and a binding enthalpy of −17.6 kcal/mol.

In order to verify the hypothesis that the rigidity of the RSL protects against the proteolytic cleavage by subtilisin, we investigated the interaction of a peptide (VCTEEY) derived from the RSL region of domain 2 of CrSPI-1 using Isothermal Titration Calorimetric (ITC) experiments (Fig. 5). Titration with the VCTEEY peptide did not resemble a hydrolytic process but a proper binding event. Other peptides (e.g., CPHTYKPVCG and LCPHTY) showed markedly different thermal profiles when injected into subtilisin (very large, constant heats of reaction, independent of the molar ratio), which could be indicative of a hydrolytic process. Nevertheless, the titration of VCTEEY showed saturation characteristics and was analyzed as a binding process that exhibited an enthalpically driven event with an affinity (K_a_) of 2×10^3^ M^−1^ (K_d_ = 500 µM) (Fig. 5). This indicates that the rigidity of the RSL peptide is not the only factor that affects the proteolytic cleavage of the inhibitor, but the sequence of the interacting peptide might also be important for inhibition. The rigidity and compactness of the domains of CrSPI-1 are due to the presence of disulphide bridges. Hence no structural changes are anticipated between native and recombinant forms. Moreover, recently we have determined the structure of domain 1 (pdb code 3PIS) which superimposed well with the CrSPI-1 domain 1 of the complex and observed no conformational changes [57].

## Discussion

### Specificity of the CrSPI-1 domains

Kazal-type serine protease inhibitors are single or multi-domain proteins with domains that usually have different specificities towards a particular protease [44]. Although the sequence of the RSL tends to be highly variable, the specificity of the overall molecule for a protease is dictated by the P1 residue. His3 is the P1 residue in the RSL of domain 1, whereas Glu48 is the P1 residue in the RSL of domain 2. Several Kazal-type inhibitors with Glu in the P1 site are known to inhibit subtilisin. For instance, the five-domain shrimp Kazal inhibitor, SPIPm2, has two subtilisin inhibiting domains with P1 Glu residues [45], and EPI1, a Kazal-like Protease Inhibitor from *Phytophthora infestans* [46], has Glu as the P1 residue. Thus, the presence of Glu in the P1 position makes the inhibitor more specific for subtilisin [45], [47]. Glu49 is buried in the S1 pocket with 8 hydrogen bonding contacts with subtilisin compared to His3, the P1 residue of domain 1, which makes only five hydrogen bonding contacts. This suggests that Glu49 in the P1 pocket of domain 2 is more specific and maintains a tight interaction with subtilisin. The RSL residues of domains 1 and 2 contribute 445 Å^2^ and 530 Å^2^ of buried surface area, respectively, which accounts for approximately 76% and 90% of the binding interfaces with subtilisin, respectively. Thus, the RSL of domain 2 contributes a greater buried area compared to the RSL of domain 1. Notably, domain 2 inhibits subtilisin with a Ki of 2.6 nM [9]. Furthermore, the ITC experiments on full-length CrSPI-1 revealed the presence of two non-identical binding sites; each domain binds one subtilisin molecule. These results suggest that domain 2 of CrSPI-1 most likely binds to a subtilisin molecule first, and then domain 1 binds a second subtilisin at a slightly lower affinity.

### CrFurin and CrSPI-1

Previously we have shown the inhibition of CrFurin by CrSPI-1 in an *ex-vivo* inhibition assay [9]. However the precise mechanism of this inhibition is not yet established. CrFurin, a homolog of subtilisin, shows specificity towards paired basic residues for cleavage [48]. Furin cleaves a wide range of proproteins at the consensus sequence Arg-Xaa-Lys/Arg-Arg-↓, and the minimal consensus sequence for Furin is Arg-Xaa-Xaa-Arg-↓. Although neither of the two domains of CrSPI-1 contains this consensus sequence, the domain 1 RSL consists of two basic residues (His3 and Lys6). Notably, Kazal-type inhibitors recognize proteases in a substrate-like manner. Furin might not cleave this RSL due to its rigid conformation and the presence of basic residues probably renders it as a substrate-like inhibitor. Several proteins have been engineered to contain a Furin consensus motif in their reactive site loops to thereby inhibit Furin. For instance, mutation of the turkey ovomucoid third domain RSL sequence from Ala-Cys-Thr-Leu18 to Arg-Cys-Lys-Arg18 or mutation of the α1-antitrypsin Portland RSL from Ala355-Ile-Pro-Met358 to Arg355-Ile-Pro-Arg358 both produced effective inhibitors for Furin [49]. These inhibitors mimic highly specific substrates. Due to the tight binding and specific rigid conformation of the RSL residues, these inhibitors are able to arrest the enzymatic reaction at the intermediate stage of hydrolysis of the peptide bond [50].

### Model of the domain 1 CrSPI-1: Furin complex

**Figure 6:**
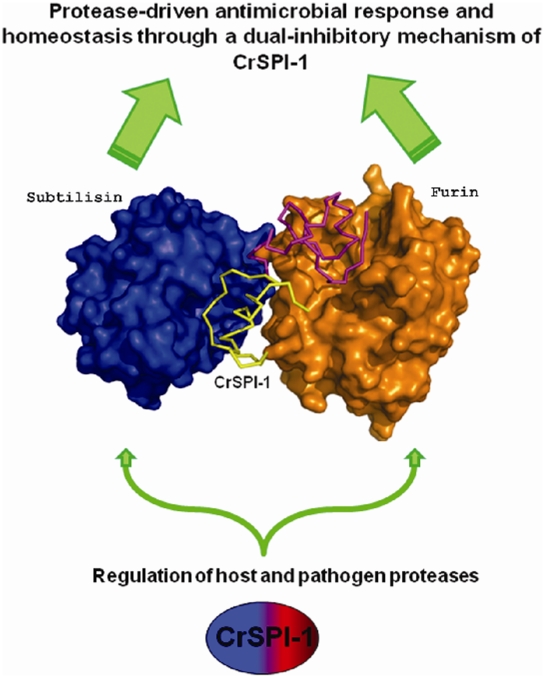
Model of Furin-CrSPI-Subtilisin heterotrimer complex. A schematic illustration on how CrSPI elicits a dual-inhibitory activity to regulate serine protease-driven antimicrobial response during acute phase innate immune response while maintaining homeostasis under naïve condition. Essentially, CrSPI employs its domain 1 (purple) to targets host's serine proteases (e.g. Furin, in brown) and its domain 2 (yellow) to target the microbial serine proteases (e.g. Subtilisin A in blue) to elicit this dual-inhibitory and regulatory mechanism.

Furin is a subtilisin-related serine protease, and a member of the proprotein convertase family. Although, subtilisin is obtained from bacteria belonging to the *Bacillus* species, Furin is considered as a eukaryotic version of subtilisin, which is ubiquitously expressed [48], . The Furin binding mode of CrSPI-1 was predicted based on the crystal structures of CrSPI-1: subtilisin and human Furin (Pdb code 1P8J). The host Furin is a homolog of the bacterial subtilisin, and these serine proteases share a sequence identity of 23% (∼40% similarity). Moreover, the structure of subtilisin superimposes on the subtilisin-like domain of Furin with an rmsd of 2 Å for 268 Cα atoms of 274 Cα atoms of subtilisin. The domain 1 CrSPI-1: subtilisin complex was superimposed onto the structure of Furin (pdb code 1P8J), and the coordinates of CrSPI-1 were copied to Furin to generate the CrSPI-1:Furin complex model (Figs. 6, S4 and S5). The RSL region from P3 (Cys1) to P3′ (Lys6) occupies the substrate binding site of human Furin. This is expected as Furins and subtilisins have highly homologous structures. However, this needs to be experimentally verified. Based on our analysis, we speculate that domain 1 of CrSPI-1 is responsible for the inhibition of CrFurin. Moreover domain 2 is shown to be an inhibitor of Subtilisin [9]. It is possible that the two domains of CrSPI-1 are products of a gene duplication event that generated a dual specificity inhibitor with one of the domains functioning as an inhibitor of host proprotein-converting subtilisin-like enzyme, CrFurin, and another domain functioning as a pathogen-specific protease (subtilisin) inhibitor.

### Possible immunomodulatory activities of CrSPI-1

The preference of CrSPI-1 domain 2 for the microbial protease subtilisin suggests that this isoform targets serine proteases of invading microbes. In fact, subtilisin is the virulence factor used by the pathogen to gain entry into host cells during an infection. Besides direct suppression of the microbial proteases, CrSPI-1 has been shown to target the host's endogenous proteases like the CrFurin. This is supported by the observation that in response to an infection, a dynamic reciprocal expression profile of the CrSPI-1 and CrFurin transcripts was observed [9]. CrSPI-1 has been proposed to regulate the serine protease-driven antimicrobial defense during the acute phase of infection while maintaining homeostasis under normal physiological conditions [9]. It has also been found to interact with microbial subtilisins as well as an endogenous C3 complement from the host, which is required for the clearance of pathogens. It is possible that CrSPI-1 serves an immunomodulatory function by interacting with host and microbial proteases through its independent domains (Fig. 6). We hypothesize that the two domains of CrSPI-1 have different substrate preferences. Domain 2 might be more potent and specific towards the bacterial protease, subtilisin, whereas domain 1 likely interacts with the host protease Furin. The structure of the CrSPI-1: subtilisin ternary complex is the first step towards an understanding of the molecular perspective of the antibacterial response while maintaining homeostasis for the host through such a dual-inhibitory mechanism. The involvement of serine proteases as the virulent factor in the entry of several microbial pathogens makes it an important target for the treatment of several infectious diseases.

## Supporting Information

Figure S1 Alignment of amino acid sequences of non-classical group I Kazal-type inhibitors AEI, CrSPI-1 domain I, domain II and a selected classical Kazal-type inhibitor OMSVP3. The sequences were aligned using CLUSTAL-W. The reactive site is denoted with an arrow. Disulfide bonds are linked as follows: α-β, II-IV, and III-VI for the non classical group I inhibitors and I-V, II-IV, and III-VI for the classical inhibitors. In nonclassical group II inhibitor family, there is an additional disulphide bridge between α and β half cystines.
(TIF)

Figure S2 Conformations of the reactive site loop (RSL). Superimposition of the reactive site loops of domain-1 (magenta), domain-2 (yellow), Eglin C (gray) and OMTKY3 (cyan). The RSLs are shown in stick representation whereas the substrate binding site of subtilisin is shown in surface representation. These figures were generated by using PyMol.
(TIF)

Figure S3 Isothermal Titration Calorimetric (ITC) curve for rCrSPI-1 titrated against subtilisin at 37°C. Each peak represents the injection of rCrSPI-1 0.2 mM into the ITC cell containing subtilisin 0.012 mM, in buffer PBS pH 7.4, 10 mM BME. A sequence of 18 injections, each injection consisting of 2 μL of ligand solution, was performed. The experimental data were fitted considering a model in which CrSPI-1 binds two Subtilisin molecules, either employing a general model based on the overall association parameters or considering two non-identical and independent binding sites in CrSPI-1 (32). Binding association constants of 2.4x10^6^ M^−1^ and 1.7x10^4^ M^−1^ were obtained from non-linear regression analysis, corresponding to dissociation constants of 0.42 and 59 μM, respectively.
(TIF)

Figure S4 Cα trace for the heterotrimer Furin-CrSPI-Subtilisin complex model. Furin and subtilisin share a sequence identity of 23%. The Furin:CrSPI-1 complex model was generated by superimposing domain-1 CrSPI-1: subtilisin complex onto the structure of Furin (pdb code 1p8j), which yielded an rmsd of 2Å for 268 Cα out of 274 Cα atoms of subtilisin. The Furin-CrSPI-1-Subtilisin heterotrimer complex was generated using the modeled Furin-CrSPI-1-domain-1 and subtilisin-CrSPI-1-domain-2 complex crystal structure.
(TIF)

Figure S5 Surface representation for Furin and Subtilisin, and backbone trace representation for CrSPI-1 of the heterotrimer model.
(TIF)

Table S1 (DOC)

Table S2 (DOC)

Table S3 (DOC)
