# Supplementary figures and images for: Structural Basis for Dual-Inhibition Mechanism of a Non-Classical Kazal-Type Serine Protease Inhibitor from Horseshoe Crab in Complex with Subtilisin
Source: PLoS One. 2011 Apr 26;6(4):e18838. doi: 10.1371/journal.pone.0018838 (PMC3082530; doi:10.1371/journal.pone.0018838)

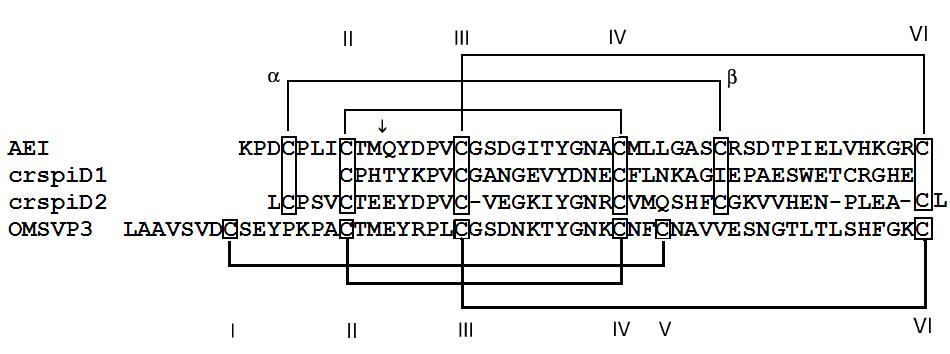

Supplement: Figure S1 — Alignment of amino acid sequences of non-classical group I Kazal-type inhibitors AEI, CrSPI-1 domain I, domain II and a selected classical Kazal-type inhibitor OMSVP3. The sequences were aligned using CLUSTAL-W. The reactive site is denoted with an arrow. Disulfide bonds are linked as follows: α-β, II-IV, and III-VI for the non classical group I inhibitors and I-V, II-IV, and III-VI for the classical inhibitors. In nonclassical group II inhibitor family, there is an additional disulphide bridge between α and β half cystines. (TIF) [file pone.0018838.s001.tif]

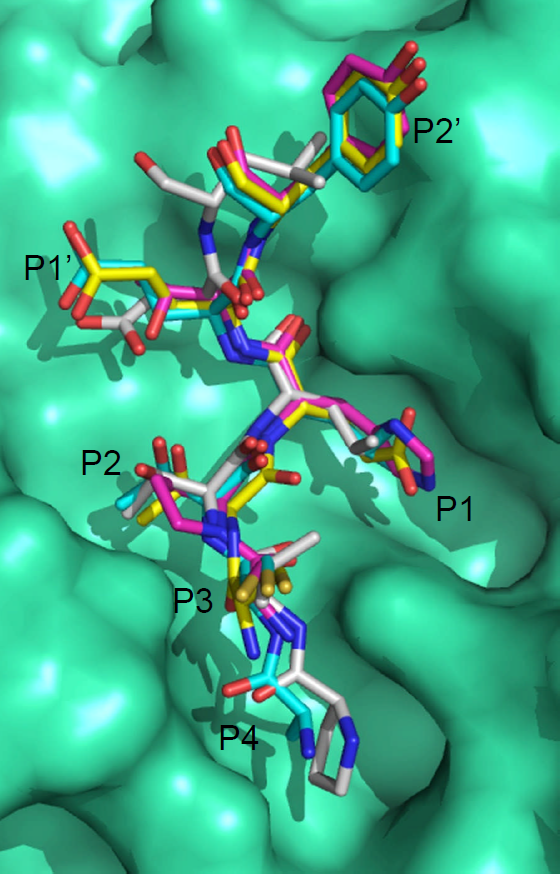

Supplement: Figure S2 — Conformations of the reactive site loop (RSL). Superimposition of the reactive site loops of domain-1 (magenta), domain-2 (yellow), Eglin C (gray) and OMTKY3 (cyan). The RSLs are shown in stick representation whereas the substrate binding site of subtilisin is shown in surface representation. These figures were generated by using PyMol. (TIF) [file pone.0018838.s002.tif]

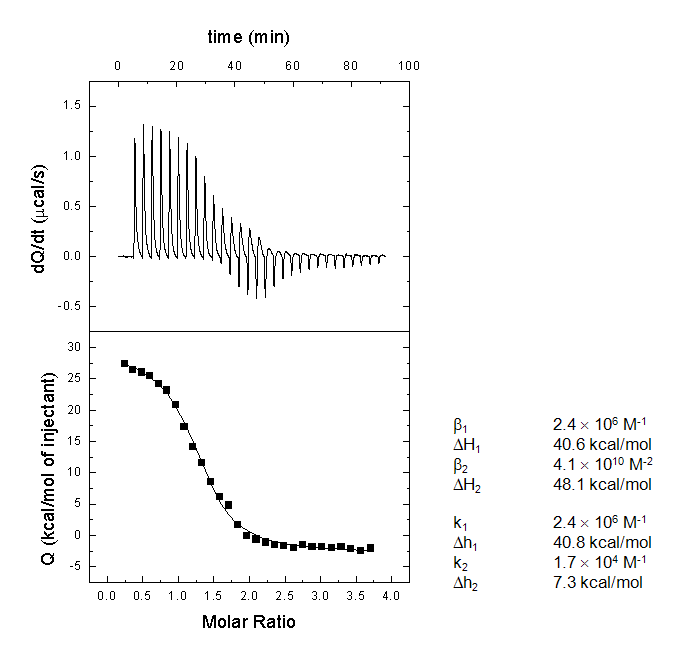

Supplement: Figure S3 — Isothermal Titration Calorimetric (ITC) curve for rCrSPI-1 titrated against subtilisin at 37°C. Each peak represents the injection of rCrSPI-1 0.2 mM into the ITC cell containing subtilisin 0.012 mM, in buffer PBS pH 7.4, 10 mM BME. A sequence of 18 injections, each injection consisting of 2 μL of ligand solution, was performed. The experimental data were fitted considering a model in which CrSPI-1 binds two Subtilisin molecules, either employing a general model based on the overall association parameters or considering two non-identical and independent binding sites in CrSPI-1 (32). Binding association constants of 2.4x10^6^ M−1 and 1.7x10^4^ M−1 were obtained from non-linear regression analysis, corresponding to dissociation constants of 0.42 and 59 μM, respectively. (TIF) [file pone.0018838.s003.tif]

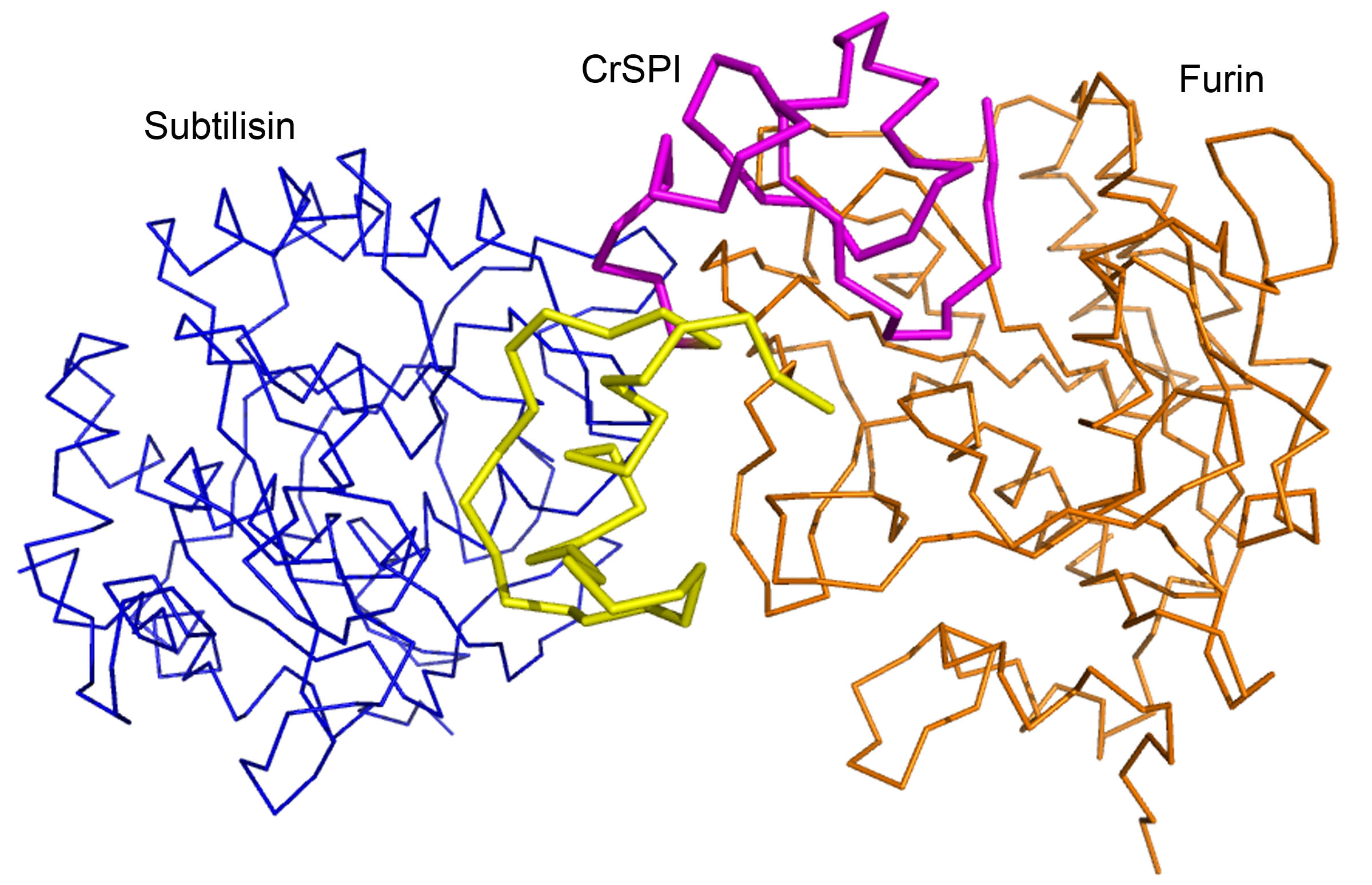

Supplement: Figure S4 — Cα trace for the heterotrimer Furin-CrSPI-Subtilisin complex model. Furin and subtilisin share a sequence identity of 23%. The Furin:CrSPI-1 complex model was generated by superimposing domain-1 CrSPI-1: subtilisin complex onto the structure of Furin (pdb code 1p8j), which yielded an rmsd of 2Å for 268 Cα out of 274 Cα atoms of subtilisin. The Furin-CrSPI-1-Subtilisin heterotrimer complex was generated using the modeled Furin-CrSPI-1-domain-1 and subtilisin-CrSPI-1-domain-2 complex crystal structure. (TIF) [file pone.0018838.s004.tif]

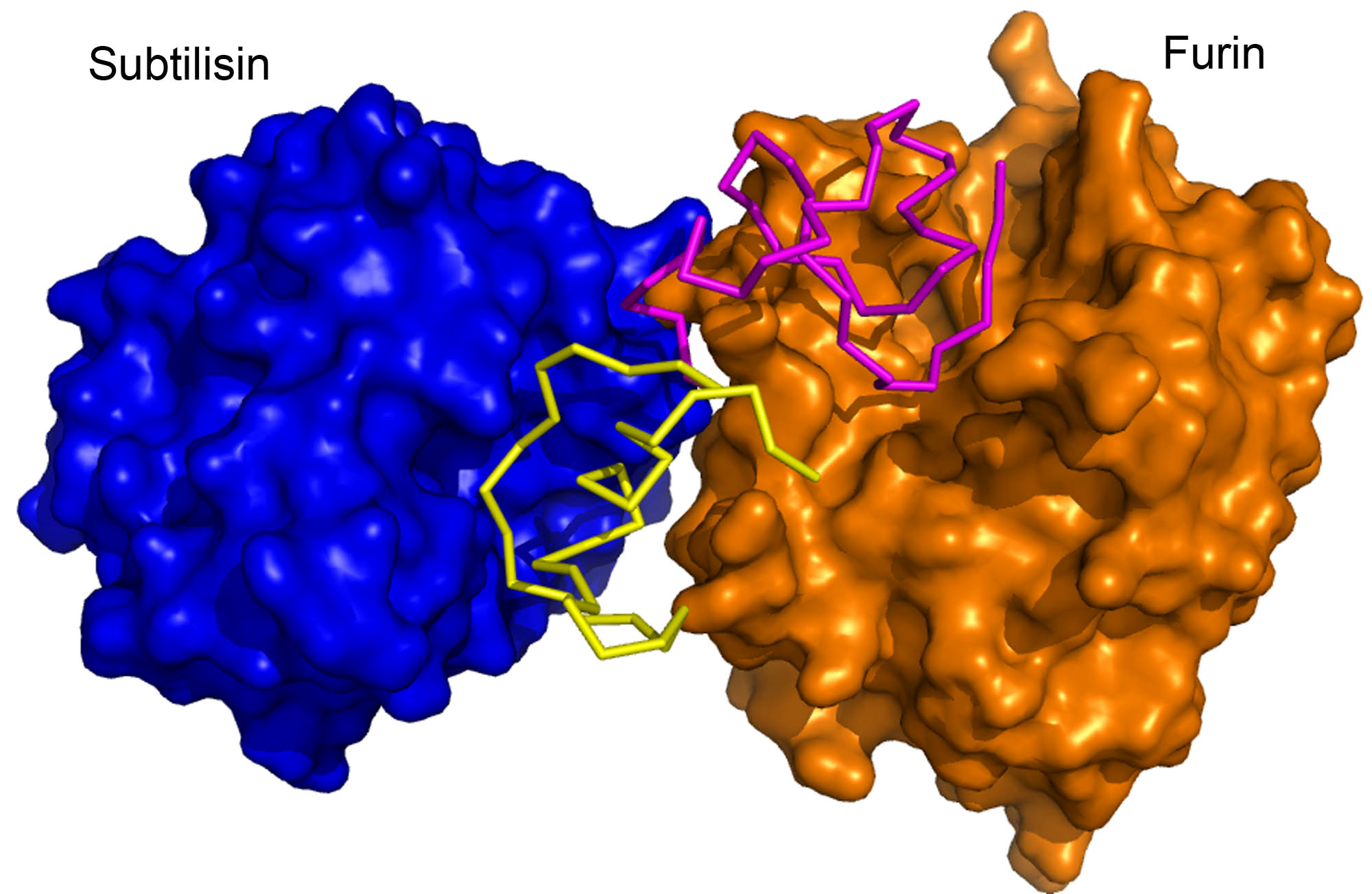

Supplement: Figure S5 — Surface representation for Furin and Subtilisin, and backbone trace representation for CrSPI-1 of the heterotrimer model. (TIF) [file pone.0018838.s005.tif]
