# Supplementary material for: Structural Basis for Dual-Inhibition Mechanism of a Non-Classical Kazal-Type Serine Protease Inhibitor from Horseshoe Crab in Complex with Subtilisin
Source: PLoS One. 2011 Apr 26;6(4):e18838. doi: 10.1371/journal.pone.0018838 (PMC3082530; doi:10.1371/journal.pone.0018838)
Supplement: Table S1 — (DOC) [file pone.0018838.s006.doc]

**Table S1.** Selected hydrogen bonding contacts between rCrSPI-1 domain-1 and subtilisin.

| **Site** | **CrSPI-1** | **Subtilisin** | **Distance (Å)** |
| --- | --- | --- | --- |
| P3 | Cys 2 NH | Gly 126 O | 3.14 |
|  | Cys 2 O | Gly 126 NH | 2.88 |
| P1 | His 4 NH | Ser 220 O^γ^ | 2.63 |
|  | His 4 N^δ1^ | Asn 154 N^δ2^ | 3.03 |
|  | His 4 O | Asn 154 N^δ2^ | 2.66 |
|  | His 4 O | Ser 220 NH | 3.28 |
|  | His 4 O | Ser 220 O^γ^ | 2.92 |
| P1’ | Thr 5 NH | Ser 220 O^γ^ | 3.34 |
| P2’ | Tyr 6 NH | Asn 217 O | 2.65 |
|  | Asp 18 O^δ1^ | Asn 154 O | 2.86 |
|  | Asp 18 O^δ2^ | Asn 154 O | 2.54 |
