# Supplementary material for: Structural Basis for Dual-Inhibition Mechanism of a Non-Classical Kazal-Type Serine Protease Inhibitor from Horseshoe Crab in Complex with Subtilisin
Source: PLoS One. 2011 Apr 26;6(4):e18838. doi: 10.1371/journal.pone.0018838 (PMC3082530; doi:10.1371/journal.pone.0018838)
Supplement: Table S2 — (DOC) [file pone.0018838.s007.doc]

| **Site** | **CrSPI-1** | **Subtilisin** | **Distance (Å)** |
| --- | --- | --- | --- |
| P4 | Val 46 O | Gly 126 NH | 3.29 |
| P1 | Glu 49 NH | Ser 220 O^γ^ | 2.75 |
|  | Glu 49 O^ε1^ | Asn 154 N^δ2^ | 2.84 |
|  | Glu 49 O^ε1^ | Asn 154 NH | 3.06 |
|  | Glu 49 O | Thr 219 NH | 3.36 |
|  | Glu 49 O | Ser 220 NH | 3.17 |
|  | Glu 49 O | Asn 154 N^δ2^ | 2.60 |
|  | Glu 49 O | Ser 220 O^γ^ | 2.84 |
|  | Glu 49 O | Asn 154 O^δ1^ | 3.32 |
| P1’ | Glu 50 NH | Ser 220 O^γ^ | 3.22 |
| P2’ | Tyr 51 NH | Asn 217 O | 2.92 |

**Table S2.** Selected hydrogen bonding contacts between rCrSPI-1 domain-2 and subtilisin
