# Supplementary material for: Structural Basis for Dual-Inhibition Mechanism of a Non-Classical Kazal-Type Serine Protease Inhibitor from Horseshoe Crab in Complex with Subtilisin
Source: PLoS One. 2011 Apr 26;6(4):e18838. doi: 10.1371/journal.pone.0018838 (PMC3082530; doi:10.1371/journal.pone.0018838)
Supplement: Table S3 — (DOC) [file pone.0018838.s008.doc]

**Table S3.** The backbone torsion angles of the Reactive Site loops of serine protease inhibitors complexed with subtilisin.

|  | CrSPI-1  Domain I torsion angles  (**Φ/ ψ)** | CrSPI-1  Domain II  torsion angles  (**Φ/ ψ)** | OMKTY3  torsion angles  (**Φ/ ψ)** | Eglin C complex  torsion angles  (**Φ/ ψ)** |
| --- | --- | --- | --- | --- |
| P4 | - | -171/-63 | -101/140 | -71/140 |
| P3 | -126/163 | 61/167 | -132/156 | -138/168 |
| P2 | -76/156 | -131/167 | -62/161 | -62/143 |
| P1 | -100/46 | -131/40 | -106/36 | -115/44 |
| P1’ | -82/146 | -91/133 | -77/134 | -96/168 |
| P2’ | -101/88 | -82/100 | -96/106 | -117/109 |
| P3’ | -122/71 | -138/68 | -142/74 | -121/112 |
